# Type II Restriction-Modification System from *Gardnerella vaginalis* ATCC 14018

**DOI:** 10.3390/pathogens9090703

**Published:** 2020-08-27

**Authors:** Aistė Bulavaitė, Indre Dalgediene, Vilma Michailoviene, Milda Pleckaityte

**Affiliations:** Institute of Biotechnology, Life Sciences Center, Vilnius University, Sauletekio al. 7, LT-10257 Vilnius, Lithuania; aiste.bulavaite@bti.vu.lt (A.B.); indre.dalgediene@bti.vu.lt (I.D.); vilma.michailoviene@bti.vu.lt (V.M.)

**Keywords:** *Gardnerella* spp., *G. vaginalis*, restriction-modification system, restriction endonuclease activity, methyltransferase activity, horizontal gene transfer

## Abstract

Intensive horizontal gene transfer may generate diversity and heterogeneity within the genus *Gardnerella*. Restriction-modification (R-M) systems and CRISPR-Cas are the principal defense tools against foreign DNA in bacteria. Nearly half of the tested *Gardnerella* spp. isolates harbored the CRISPR-Cas system. Several putative R-M systems of *Gardnerella* spp. strains were identified in the REBASE database. However, there was no experimental evidence for restriction endonuclease (REase) activity in the isolates. We showed that *G. vaginalis* strain ATCC 14018 contains the REase R.Gva14018I, which recognizes GGCC and most probably generates blunt ends on cleavage. Bioinformatics evidence and the activity of recombinant methyltransferase M.Gva14018I in vivo indicate that ATCC 14018 possesses a HaeIII-like R-M system. The truncated R.Gva14018I-4 lacking the C-terminal region was expressed in *Escherichia coli* and displayed wild-type REase specificity. Polyclonal antibodies against R.Gva14018I-4 detected the wild-type REase in the cell lysate of ATCC 14018. The cofactor requirements for activity and bioinformatics analysis indicated that R.Gva14018I belongs to the PD-(D/E)XK family of REases. The REase-like activity was observed in 5 of 31 tested *Gardnerella* spp. strains, although none of these matched the DNA digestion pattern of R.Gva14018I.

## 1. Introduction

Vaginal anaerobic dysbiosis, bacterial vaginosis (BV), affects women’s self-esteem and sex life [1] and is associated with poor reproductive and obstetric sequelae [2,3,4]. An indicator of BV is the presence of ‘clue cells,’ which are desquamated vaginal epithelial cells coated with a biofilm [5,6]. The biofilm matrix is built of *Gardnerella* spp. and other less numerous bacterial species are embedded within the matrix [6]. *Gardnerella* spp. are considered as key in the progression of anaerobic dysbiosis because of their predominance in BV and exceptional virulence potential compared to other BV-associated bacteria [5,7,8,9]. However, *Gardnerella* spp. are often found in high numbers in asymptomatic women with *Lactobacillus*-dominated vaginal microbiota [10,11,12]. Different clinical phenotypes suggest heterogeneity and diversity within the genus *Gardnerella* [13,14,15,16]. Research groups using distinct genetic targets clustered *Gardnerella* isolates into four clades/subgroups with different genomic properties [13,17]. In 2019, the *Gardnerella* taxonomic description was amended based on comparisons of whole-genome sequences and MALDI-TOF mass spectrometry analysis, resulting in four species (*G. vaginalis*, *G. leopoldii*, *G. swidsinskii*, and *G. piotii*) and nine ‘genome species’ [18]. It is likely that named *Gardnerella* species and ‘genome species’ are specifically associated with BV due to differences in their virulence potential. The expression profiles of virulence-associated genes can be affected by interactions among *Gardnerella* species/subgroups [19] and between *Gardnerella* and other bacterial species [20,21] and the host.

High variability in gene contents of *Gardnerella* spp. isolates may originate from horizontal gene transfer realized via the acquisition of mobile genetic elements, prophages, and foreign DNA from the surrounding medium [22,23]. The principal prokaryotic defense tools against foreign invading DNA consist of clustered regularly interspaced short palindromic repeats and their associated Cas proteins (CRISPR-Cas) and restriction-modification system [24,25,26]. The CRISPR-Cas system has been experimentally identified in approximately 50% of analyzed *Gardnerella* clinical isolates [27]. The spacer contents in the CRISPR arrays suggest that the loci in CRISPR-positive isolates were active [27].

Restriction-modification (R-M) systems are widespread among prokaryotes; an average of two R-M systems are found in the bacterial genome [28]. The abundance of R-M systems varies among microorganisms, from a single system in the majority of *Francisella noatunensis* and *Bacillus anthracis* strains, to the large number of systems in *Neisseria gonorrhoeae* and *Helicobacter pylori* [29,30,31] (rebase.neb.com/rebase/rebase.html), and the complete lack of systems in *Anaplasma*, *Rickettsia*, *Coxiella*, *Borrelia*, and *Chlamydia* [32] (rebase.neb.com/rebase/rebase.html). The R-M system consists of two enzymatic activities that ensure discrimination between self and nonself DNA: restriction endonuclease (REase) and methyltransferase (MTase) [33,34]. Microorganisms with smaller genomes often possess REases that recognize a short 4-bp target sequence. Those with large genomes tend to have enzymes that recognize longer sequences and minimize accidental breaks of the host genome [32]. R-M systems are classified into four types and their respective subtypes on the basis of the mechanism of target recognition, genetic and molecular organization, and cofactor requirements [33]. Several putative R-M systems (type I–III) of *Gardnerella* spp. strains were identified in the REBASE database [35] (rebase.neb.com/rebase/rebase.html). However, there was no data on REase activity in *Gardnerella* spp. isolates. Here, we identified and characterized the R-M system encoded in the genome of *Gardnerella vaginalis* strain ATCC 14018. We produced *G. vaginalis*–derived recombinant REase R.Gva14018I and characterized its enzymatic activity. A collection of 31 *Gardnerella* spp. isolates were assessed for the REase activity.

## 2. Results

### 2.1. R-M Systems in Gardnerella spp. and Detection of Restriction Endonuclease Activity

**Figure 1:**
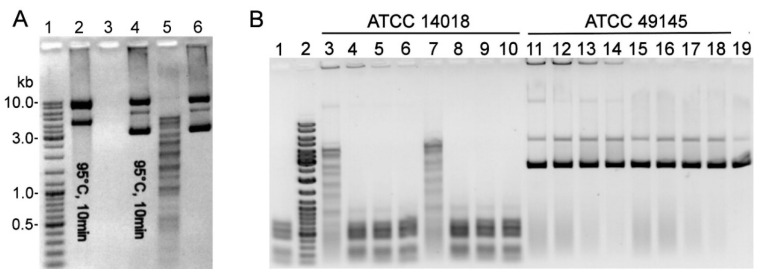
Detection of enzymatic activity. (**A**) Enzymatic activity in the cell-free sTSB culture medium (optical density (OD_600_ ) reached 0.65) of *G. vaginalis* ATCC 14018; 2 µL of culture medium (lanes 4, 5) or 1 unit of DNaseI (lanes 2, 3) was incubated with pBR322 for 45 min at 37 °C. Lanes 2, 4: the reaction mixture was preincubated at 95 °C for 10 min; lane 6: untreated pBR322; lane 1: DNA size standard Gene Ruler™ Ladder Mix (Thermo Fisher Scientific). (**B**) Enzymatic activity in the cell lysates of *G. vaginalis* ATCC 14018 and ATCC 49145 strains. Various amounts of the PBS-diluted sonicated (lanes 3–6, 11–14) and centrifuged (lanes 7–10, 15–18) cell lysates were added to the pBR322-containing reaction mixture and incubated for 10 min at 20 °C (lanes 3, 7, 11, 15) or 2 h at 37 °C (lanes 4–6, 8–10, 12–14, 16–18). Lane 1: pBR322 treated with the cell-free *G. vaginalis* ATCC 14018 culture supernatant (OD_600_ = 0.6); lane 2: DNA size standards Gene Ruler™ Ladder Mix; lane 19: untreated pBR322.

Nuclease activity was observed in cell-free sTSB medium (TSB supplemented with 0.15% (*w*/*v*) soluble starch and 2% (*v*/*v*) horse serum) collected after 24 h of *G. vaginalis* ATCC 14018 cultivation (Figure 1A). A particular array of DNA fragments was produced by incubating pBR322 with the cell-free culture supernatants and clarified cell lysates from serum-free sBHI (BHI supplemented with 2% (*w*/*v*) gelatin, 0.5% yeast extract (*w*/*v*), and 0.1% soluble starch (*w*/*v*)) culture medium, suggesting a restriction of endonuclease (REase) activity (Figure 1B).

**Figure 2:**
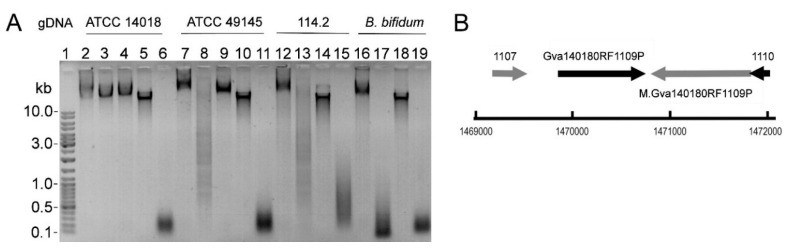
(**A**) The susceptibility of gDNAs to cleavage by endonuclease in *Gardnerella* spp. cell-free culture supernatants. gDNAs from *G. vaginalis* ATCC 14018 and ATCC 49145, *Gardnerella* spp. 114.2, and *Bifidobacterium bifidum* were incubated with culture supernatants from strains ATCC 14018 (lanes 3, 8, 13, 17) and 49145 (lanes 4, 9) as described in Methods. Lane 1: Gene Ruler™ Ladder Mix; lanes 2, 7, 12, 16: respective gDNA treated with fresh sBHI medium (negative control); lanes 5, 10, 14, 18: untreated respective gDNA; lanes 6, 11, 15, 19: respective gDNAs treated with the *S. pneumoniae* culture supernatant. (**B**) The chromosome position of the DNA locus coding for putative R-M system proteins in *G. vaginalis* ATCC 14018 (http://tools.neb.com/genomes/view.php?enzname=Gva14018ORF1109P). GAVG_1107 conserved hypothetical protein (1,469,182–1,469,529 nt); GAVG_1108 conserved hypothetical protein (Gva14018ORF1109P, 1,469,855–1,470,751 nt); GAVG_1109 DNA methyltransferase (M.Gva14018ORF1109P, 1,470,803–1,471,804 nt); and GAVG_1110 putative transcriptional regulator (1,471,801–1,472,019 nt).

The samples of cell-free culture supernatants withdrawn consecutively during strain cultivation displayed an increasing DNA cleavage activity (Figure S1). The genomic DNA (gDNA) extracted from ATCC 14018 was resistant to cleavage by the respective supernatant, whereas gDNAs from *G. vaginalis* ATCC 49145 and *Gardnerella* spp. 114.2 strains [12] were susceptible to cleavage (Figure 2A). The results were consistent with the activity of a bacterial restriction-modification (R-M) system comprised of two enzymes: REase combined with methyltransferase (MTase), which modifies bacterial DNA to protect from the respective REase digestion [34].

The putative R-M systems of three *Gardnerella* spp. strains are presented in the REBASE database: *G. swidsinskii* strain GV37 [18,36] has two Type I systems, one Type II system, and one Type IV system; *G. vaginalis* strain ATCC 49145 has one each of Type I and Type III systems; and *G. vaginalis* strain ATCC 14018 has one complete Type II system with the indicated GGCC target sequence. No endonuclease activity was detected using a Dam and Dcm methylated substrate in the cell-free culture medium of ATCC 49145 (Figure 1B), GV37, and 35 other *Gardnerella* isolates of different subgroups obtained from the study [12] (data not shown). The cell lysates of ATCC 49145 (Figure 1B), GV37, and 31 *Gardnerella* spp. isolates were assayed for endonuclease activity using both Dam and Dcm methylated and non-methylated substrates. Specific arrays of DNA fragments were generated by adding cell lysates of five isolates with presumed REase-like activity (Figure S2). Substrates with both methylation patterns were affected by clarified cell lysates of strains 47.3 and 105.1 (both of clade 1), whereas lysates of strains 63.2, 78.1, and 86.3 (all of clade 2) digested only non-methylated DNA (Figure S2). The distinct patterns of DNA fragments produced by REases of these five strains did not match that digested by the ATCC 14018 enzyme.

The DNA fragments produced by pBR322 digestion of the ATCC 14018 supernatant were cloned into the pJET1.2 vector and subjected to sequencing. The results identified the GGCC sequence as a cleavage target. Incubation of the DNA fragments with blunting enzyme (see subchapter *Determination of REase cleavage site*) before ligation retained the GGCC motif between the ligated fragments. The blunting enzyme has a proofreading activity assuring the removal of 3′-overhangs and fill-in of 5′-overhangs. The retaining of GGCC strongly suggested the blunt-ended cleavage GG↓CC by the REase. The detected REase target sequence was consistent with that presented in the REBASE database (http://rebase.neb.com/cgi-bin/seqget?Gva14018ORF1109P).

The DNA locus predicted to encode the R-M system of *G. vaginalis* ATCC 14018 strain was designated *gva14018I*. The sequences coding for REase and MTase are in opposite orientations (Figure 2B). The *gva14018IR* locus codes for the putative Type II restriction endonuclease R.Gva14018I protein of 34 kDa. The locus *gva14018IM* codes for the putative methyltransferase M.Gva14018I protein of 38 kDa. The other 36 *Gardnerella* spp. isolates and ATCC 49145 strain were negative for *gva14018IR* and *gva14018IM* in the gene-specific PCR assays (Figure S3).

### 2.2. Cloning and Expression of gva14018IM and gva14018IR in E. coli

**Figure 3:**
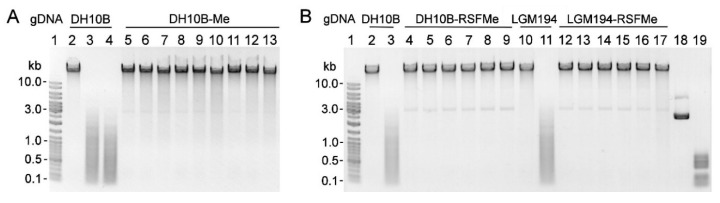
Susceptibility of *E. coli* gDNA to cleavage by REase in *G. vaginalis* ATCC 14018 culture supernatant. (**A**) gDNAs extracted from *E. coli* DH10B (lanes 2–4) and three individual DH10B-Me colonies (lanes 5–13) were incubated with fresh sBHI medium (negative control) (lanes 2, 5, 8, 11), *G. vaginalis* ATCC 14018 culture (OD_600_ = 0.6) supernatant (lanes 3, 6, 9, 12), and BsuRI (lanes 4, 7, 10, 13). (**B**) gDNAs extracted from *E. coli* DH10B (lanes 2–3), three individual DH10B-RSFMe colonies (lanes 4–9), LMG194 (lanes 10–11), and three individual LMG194-RSFMe colonies (lanes 12–17) were incubated with fresh sBHI medium (negative control) (lanes 2, 4, 6, 8, 10, 12, 14, 16) and *G. vaginalis* ATCC 14018 culture (OD_600_ = 0.6) supernatant (lanes 3, 5, 7, 9, 11, 13, 15, 17). Lanes 18, 19: untreated pBR322 and pBR322 incubated with ATCC 14018 culture supernatant, respectively. (A, B) Lane 1: Gene Ruler™ Ladder Mix.

The amplified *gva14018IM* gene (GenBank accession no. MN938913) was cloned downstream of the tetracycline resistance gene promoter in plasmid pACYC184. The recombinant plasmid was transformed into *E. coli* DH10B, ER2566, and LMG194 strains. The recombinant MTase ensured protection against the wild-type REase digestion: gDNAs extracted from the pACYC-Me–transformed *E. coli* strains were protected from cleavage by the *G. vaginalis* ATCC 14018 culture supernatant, whereas gDNAs from the non-transformed *E. coli* strains were susceptible to cleavage (Figure 3A). Transformed strains that displayed a high level of protection were selected for subsequent cloning of REase (Table S1).

The amplified *gva14018IR* gene was cloned into pJET1.2, pET28a(+), and pBAD vectors, and transformed into *E. coli* strains that displayed either high-level protection or no protection against cognate REase. DNA sequencing revealed the mutations, which most likely appeared during gene cloning. Each mutation resulted in changes in the amino acid sequence of the R.Gva14018I protein. The mutation-free *gva14018IR* gene was obtained by cloning the PCR product into the pUC57 vector, with subsequent transformation into the methylation-proficient DH10B-Me strain. Recombinant plasmids were selected that harbored the *gva14018IR* gene in opposite orientation to the lacZ promoter and the distant β-lactamase promoter. It was expected that reduced transcription of the target gene from the major annotated promoters would result in the mutation-free gene. However, subcloning of *gva14018IR* into the expression vectors pET28a(+) and pBAD did not yield the mutation-free gene even in the presence of glucose, which represses the activity of the respective promoters. We speculated that the synthesized amount of M.Gva14018I was too low to perform complete methylation in vivo, and an insufficient level of protection against the wild-type REase may promote the in vivo selection of colonies harboring mutated *gva14018IR*. To increase the amount of MTase, we constructed plasmid pRSFori-Me, which has a higher copy number than pACYC-Me. The gDNAs extracted from DH10B-RSFMe and LMG194-RSFMe were resistant to cleavage by cognate REase (Figure 3B). However, subcloning of *gva14018IR* into the pBAD expression vector with subsequent transformation in DH10B-RSFMe strain resulted in the occurrence of mutations in *gva14018IR*.

### 2.3. Expression of Mutated gva14018IR Genes in E. coli

**Figure 4:**
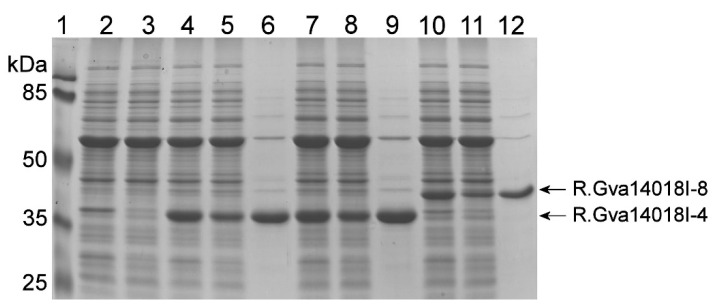
SDS-PAGE analysis of R.Gva14018I mutants produced in *E. coli* ArcticExpress (DE3). R.Gva14018I-4–expressing cell lysates (lanes 4, 7), clarified lysates (lanes 5, 8), and captured His-tagged R.Gva14018I-4 from an IMAC column (Zymo Research) (lanes 6, 9). R.Gva14018I-8–expressing cell lysates (lane 10), clarified lysates (lane 11), and captured His-tagged R.Gva14018I-8 (lane 12). As controls, lysates (lane 2) and clarified lysates (lane 3) of *E. coli* cells transformed with pET28(+) vector were loaded on the gel. Lane 1: prestained Protein Molecular Weight Marker (Thermo Fisher Scientific). Arrows indicate the migration positions of R.Gva14018I mutants.

Two mutated *gva14018IR* genes were selected for further experiments. The first gene variant had one nucleotide deletion, which generated a fragment encoding the truncated protein comprising the first 259 amino acids of the wild-type REase (298 amino acids) followed by WKGEMLYMND from a mutation-generated frameshift. This gene was named *gva14018IR-4* (GenBank accession number MN738700). The second gene variant *gva14018IR-8* encoded the REase protein containing the W129R amino acid substitution (GenBank accession no. MN938912). Both gene variants were cloned into pET28a(+) and transformed into *E. coli* strains Tuner (DE3), ER2566, and ArcticExpress (DE3). The *E. coli* strains selected for transformation did not possess a plasmid harboring the *gva14018IM* gene. R.Gva14018I-4 and R.Gva14018I-8 were expressed in *E. coli* as N-terminal His-tagged proteins with apparent molecular weights slightly higher than calculated (33 and 36 kDa, respectively) (Figure 4). *E. coli* ArcticExpress (DE3) was selected for recombinant protein production because it has low endogenous nuclease activity (Figure 4).

**Figure 5:**
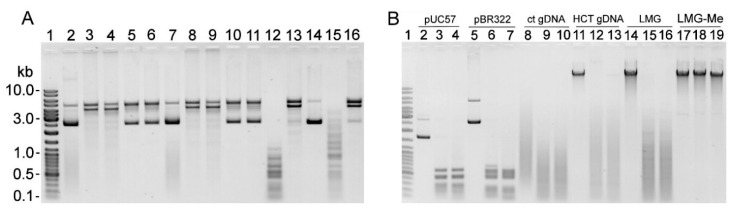
REase activity of R.Gva14018I mutants. (**A**) pBR322 incubated with *E. coli* ArcticExpress (DE3) expressing R.Gva14018I-4: cell lysates (lanes 3, 4), clarified lysates (lanes 8, 9), and crude protein R.Gva14018I-4 preparations (lane 15). pBR322 incubated with R.Gva14018I-8-expressing cell lysates (lanes 5, 6), clarified lysates (lanes 10, 11), and crude protein R.Gva14018I-8 preparations (lane 16). Lanes 2, 7: pBR322 treated with cell lysate and clarified lysate of *E. coli* cells transformed with pET28(+) vector, respectively. Lanes 12, 13: pBR322 treated with undiluted (OD_600_ = 0.6) and a 10-fold diluted *G. vaginalis* ATCC 14018 cell-free supernatant, respectively. Lane 14: untreated pBR322. (**B**) REase activity of the purified recombinant R.Gva14018I-4 and wild-type REase in cell-free supernatant of ATCC 14018. The substrates pUC57, pBR322, gDNAs from calf thymus, human cell line HCT116, *E. coli* LMG194, and *E. coli* LMG194-Me were incubated with the supernatant (lanes 3, 6, 9, 12, 15, 18) or R.Gva14018I-4 (lanes 4, 7, 10, 13, 16, 19). Lanes 2, 5, 8, 11, 14, 17: untreated respective DNA substrate. (A, B) Lane 1: Gene Ruler™ Ladder Mix.

Cell lysates and crude protein preparations exhibited R.Gva14018I-specific activity (Figure 5A). R.Gva14018I-4 displayed higher REase activity than R.Gva14018I-8 and was selected for further experiments. A fraction of soluble R.Gva14018I-4 obtained after the induction of protein synthesis in ArcticExpress (DE3) at 12 °C was subjected to purification via single-step Ni-affinity chromatography (Figure 5).

### 2.4. Polyclonal Antibodies against Recombinant R.Gva14018I-4 also Detected R.Gva14018I

**Figure 6:**
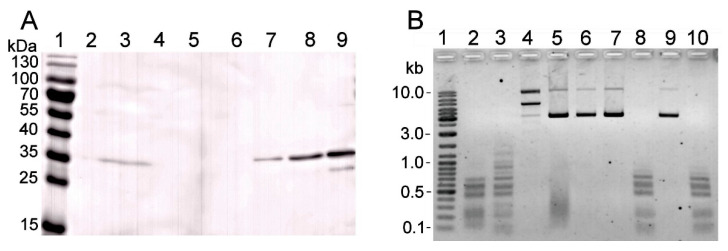
Identification of wild-type and recombinant REases via immunoblot (**A**) and REase activity towards the DNA substrate (**B**). (**A**) Immunoblot with purified R.Gva14018I-4 pAbs. Lane 3: cell lysate of ATCC 14018 (approx. 0.3 mg of the total protein); lane 5: cell lysate of ATCC 49145 (approx. 0.4 mg of the total protein); lanes 7–9: 0.2, 0.5, and 1 µg of recombinant R.Gva14018I-4. Lanes 2, 4, 6: empty lanes; lane 1: PageRuler Prestained Protein Ladder (Thermo Fisher Scientific #26616). (**B**) Cell lysates subjected to immunoblot analysis (**A**) were tested for REase activity via a DNA agarose gel electrophoresis. pBR322 was incubated with 10-fold serially diluted cell lysates of strain ATCC 14018 (lanes 2–4) and ATCC 49145 (lanes 5–7), purified R.Gva14018I-4 (lane 8), PBS (lane 9), and cell-free culture supernatant of ATCC 14018 (lane 10). Lane 1: Gene Ruler™ Ladder Mix.

To detect the wild-type REase R.Gva14018I produced by *G. vaginalis* cells, the polyclonal antibodies (pAbs) were raised by immunizing mice with purified R.Gva14018I-4. The pAb immunoprecipitates of R.Gva14018I-4 were subjected to mass spectrometry analysis. The major peak in the HPLC/ESI-MS chromatogram corresponded to 33,075.20 Da (Figure S4). The detected molecular mass of R.Gva14018I-4 was in good agreement with the mass deduced from its amino acid sequence (33,074.29 Da without the N-terminal methionine; https://web.expasy.org/protparam/). Immunoblot analysis of cell lysates of *G. vaginalis* strain ATCC 14018 revealed a band in the range of 35–40 kDa using pAbs against R.Gva14018I-4 as the primary antibody (Figure 6A). No bands were visualized via immunoblot analysis of the cell lysates of *G. vaginalis* strain 49145. REase activity was detected by immunoblot only in the ATCC 14018 cell lysate (Figure 6B).

### 2.5. Characterization of the R.Gva14018I-4 Protein

The specificities of wild-type and recombinant REases were compared by digesting DNA substrates with ATCC 14018 culture supernatant and purified R.Gva14018I-4 (Figure 5B). Similar arrays of DNA fragments were obtained after the digestion of pUC57 and pBR322 plasmids. Incubation of pBR322 with the supernatant yielded an extra band, which was most likely a product of incomplete DNA cleavage (Figure 5B, lane 6). Both REases digested gDNAs from calf thymus, HCT116 human cell line, and *E. coli* LMG194, whereas gDNA from the LMG194-Me strain was resistant to cleavage.

To determine the target sequence of R.Gva14018I-4, a single-site PCR substrate was digested, and the DNA fragments were cloned into pJET1.2 plasmid and then sequenced. The results showed that R.Gva14018I-4 cleaved the GGCC sequence. Thus, both the wild-type and recombinant *G. vaginalis* 14018 REases recognized the same target sequence and were sensitive to the methylation pattern generated by recombinant M.Gva14018I.

**Figure 7:**
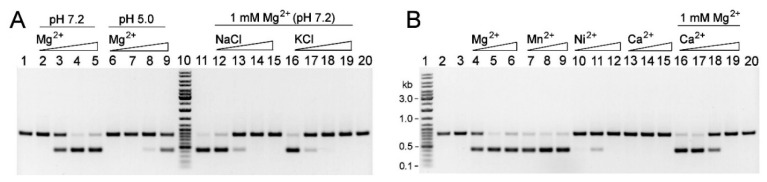
Effects of cofactors, pH, and salt concentrations on R.Gva14018I-4 activity. (**A**) Single-site PCR substrate was digested with purified R.Gva14018I-4 in the presence of increasing amounts of Mg^2+^ (0, 0.1, 1, 10 mM) at pH 7.2 (lanes 2–5) and pH 5.0 (lanes 6–9). The reaction mixture containing 1 mM Mg^2+^ at pH 7.2 (lane 11) was supplemented with 50, 100, 150, and 200 mM NaCl or KCl to test the effects of salt concentration (lanes 12–19). Lanes 1, 20: untreated substrate. (**B**) Single-site PCR substrate cleavage at pH 7.2 in the presence of increasing amounts (0.1, 1, 10 mM) of Mg^2+^ (lanes 4–6), Mn^2+^ (lanes 7–9), Ni^2+^ (lanes 10–12), and Ca^2+^ (lanes 13–15). R.Gva14018I-4 was incubated with DNA in reactions supplemented with 1 mM Mg^2+^ and increasing amounts (0, 0.1, 1, 10 mM) of Ca^2+^ (lanes 16–19). Lanes 2, 20: untreated substrate; lane 3: no divalent metal ions added. Lane 10 (**A**) and lane 1 (**B**): Gene Ruler™ Ladder Mix.

The single-site PCR substrate was used to evaluate the effects of pH and salt and divalent cation concentrations on purified R.Gva14018I-4 activity. At neutral pH 7.2, R.Gva14018I-4 displayed maximum activity in the presence of 1–10 mM Mg^2+^, but the activity significantly decreased at pH 5.0 (Figure 7A). At neutral pH, increasing concentrations of NaCl or KCl from 0–200 mM inhibited REase activity. Divalent metal ions Mg^2+^ and Mn^2+^ were preferred cofactors. R.Gva14018I-4 displayed maximum activity in the presence of 1 mM Mg^2+^, whereas the addition of 0.1 or 10 mM Mg^2+^ resulted in incomplete substrate digestion. The optimum concentration of Mn^2+^ in the reaction mixture was 0.1–10 mM. Low REase activity was observed at 1 mM Ni^2+^ (Figure 7B). The addition of Ca^2+^ (0.1–10 mM) to the reaction mixture did not induce REase activity. In the presence of 1 mM Mg^2+^, increasing Ca^2+^ concentrations inhibited substrate digestion (Figure 7B). Similar effects of pH and salt and divalent cation concentrations on REase activity were observed with pUC57, which has multiple R.Gva14018I-4 recognition sites (Figure S5).

### 2.6. Bioinformatics Analyses of M.Gva14018I, R.Gva14018I, and R.Gva14018I-4

M.Gva14018I was used as a query sequence for a BLASTp search which revealed multiple bacterial proteins characterized as C-5 cytosine-specific DNA methyltransferases (pfam00145) that are often found with HaeIII family (pfam09556) REases. HaeIII cleaves the double-stranded sequence GGCC and generates blunt ends (UniprotKB entry O68584). Its cognate methyltransferase modifies cytosine to 5-methylcytosine (GGm5CC) on both DNA strands (UniprotKB P20589). The gDNA extracted from DH10B-Me was resistant to cleavage by BsuRI (Figure 3A); BsuRI does not cleave the methylated target GGm5CC sequence [37]. These combined results suggest that M.Gva14018I modifies the first cytosine in the GGCC sequence.

**Figure 8:**
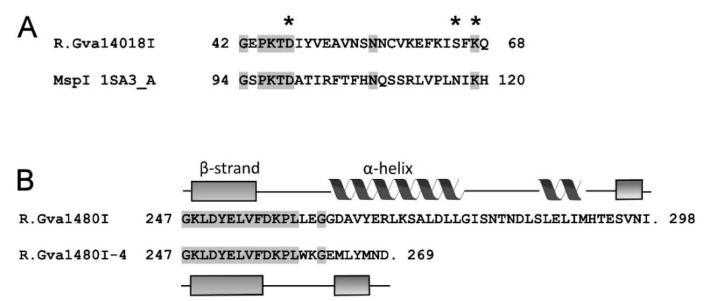
Bioinformatics analysis of R.Gva1408I sequence motifs. (**A**) Sequence alignment between the active site of MspI [40] and the putative active-site region of R.Gva1408I. Identical residues are shaded in gray. R.Gva1408I sequence similarity with MspI (PDB ID 1SA3 chain A) was determined using HHpred [41]. The conserved amino acids D47, S65, and K67 of the predicted PD-(D/E)XK active site of R.Gva1408I are marked with asterisks [39,40]. (**B**) Sequence alignment of the C-terminus of wild-type R.Gva14018I and truncated R.Gva14018I-4. Identical residues are shaded in gray. PSIPRED [42] was used to predict the secondary structure elements of the molecules.

A BLASTp search with the R.Gva14018I sequence found 39% identity and 57% similarity with the restriction endonuclease BspRI (GenBank CBE66553.1), a single protein with a known function among multiple matches. BspRI is an experimentally determined Type IIP REase that cleaves DNA at the sequence GGCC to produce blunt-end fragments [38]. Sequence analysis of R.Gva14018I using the PDEXK server [39] showed a probability of 0.95 that the nuclease belongs to the PD-(D/E)XK superfamily. The HHpred analysis (https://toolkit.tuebingen.mpg.de/tools/hhpred) revealed that the N-terminal region (amino acids 38–104) is similar to the amino acid 90–156 region of MspI. This result suggests that the active site of R.Gva14018I is located between 38–104 amino acids [40]. Amino acids D47, S65, and K67 most likely constitute part of the active site. Amino acid S65 conforms to neither D/E in the PD-(D/E)XK motif, nor N117 in the respective motif of MspI. However, BsuFI displays S254 in the same position [40]. The presence of the first 259 amino acids at the N-terminus presumes that R.Gva14018I-4 contains an intact catalytic center. Secondary structure prediction of wild-type and mutant R.Gva14018I was performed by the PSIPRED server (http://bioinf.cs.ucl.ac.uk/psipred). The truncated R.Gva14018I-4 protein lacked the C-terminal alpha helix (263–277 amino acids) (Figure 8). We speculate that the difference in secondary structure might affect the enzyme conformation and activity. The absence of the C-terminus might result in inefficient DNA cleavage by R.Gva14018-4 compared to that of the wild-type REase. This presumption is supported by the fact that *E. coli* cells transformed with a plasmid bearing the mutant gene *gva14018IR-4* survived even in the absence of cognate MTase, whereas the high toxicity of wild-type R.Gva14018I endonuclease in *E. coli* impairs its gene cloning.

## 3. Discussion

In this study, we identified and characterized the R-M system from *G. vaginalis* strain ATCC 14018. The genes predicted to encode the R-M system were indicated in the REBASE database. The open reading frames encoding the putative REase and MTase were on complementary strands interspaced by 53 bp and oriented toward each other. A putative transcription regulator (GenBank BAQ33762.1) is encoded immediately upstream of the MTase. The REase and MTase are the first characterized R-M enzymes from the genus *Gardnerella*. *G. vaginalis* ATCC 14018 possesses the REase R.Gva14018I and its cognate MTase. R.Gva14018I cleaves the GGCC sequence, most probably generating blunt ends. A BLASTp search identified similarity with the characterized REase BspRI, which cleaves the GG↓CC sequence. Experimental evidence indirectly demonstrated that M.Gva14018I modifies the first cytosine residue in the GGCC sequence. Moreover, similarity searches for M.Gva14018I showed many C-5 cytosine-specific DNA MTases, which generate GGm5CC methylation on both DNA strands. *G. vaginalis* ATCC 14018 possesses a HaeIII-like R-M system, which is widespread among bacterial species [38,40,43]. A HaeIII-like restriction endonuclease activity is an effective tool against foreign DNA because the short cleavage site frequently occurs [43].

The gene encoding MTase was cloned and expressed in *E. coli*. Recombinant M.Gva14018I was active in vivo. Cloning and expression of R.Gva14018I in *E. coli* cells was challenging due to toxicity, which is common for REases [38,44]. R.Gva14018I mutants were successfully expressed in *E. coli*. The truncated R.Gva14018I-4 protein lacking the C-terminal region demonstrated the highest REase activity. The same target specificity was observed for purified R.Gva14018I-4 and wild-type R.Gva14018I from the cell-free lysate of *G. vaginalis* ATCC 14018. R.Gva14018I-4 requires Mg^2+^ for its enzymatic activity; Mn^2+^ can substitute as a cofactor for Mg^2+^, whereas Ca^2+^ cannot. The same cofactor requirements are characteristic of Type II REases of the PD-(D/E)XK family, whereas REases of the GIY-YIG and HNH families use various metal cations and Ca^2+^ as cofactors [45,46]. Our bioinformatics analysis strongly supports the prediction that R.Gva14018I belongs to the PD-(D/E)XK family of REases.

The REase activity was detected in *G. vaginalis* cell lysate and cell-free growth medium. REase activity in cell-free growth medium is presumably due to cell death and decomposition as the DNA cleavage activity increased over the time course of cultivation. We were unable to purify wild-type R.Gva14018I from cell-free extracts or cell-free growth medium. Polyclonal antibodies against R.Gva14018I-4 detected the wild-type R.Gva14018I REase in the cell lysate of *G. vaginalis* strain ATCC 14018. The pAbs did not react with the cell lysate of strain ATCC 49145, which did not display REase activity. The apparent molecular mass of the protein recognized by pAbs was slightly larger than the predicted 34 kDa for R.Gva14018I. R.Gva14018I-4 produced a band that was observed at a higher position on the gel than that of its calculated molecular mass, although mass spectrometry of the pAb-immunoprecipitated complexes showed good agreement between calculated and experimental values for R.Gva14018I-4 molecular mass. Immunoprecipitation of wild-type R.Gva14018I from the cell lysate was not successful, possibly due to the high amount of interfering compounds.

**Figure 9:**
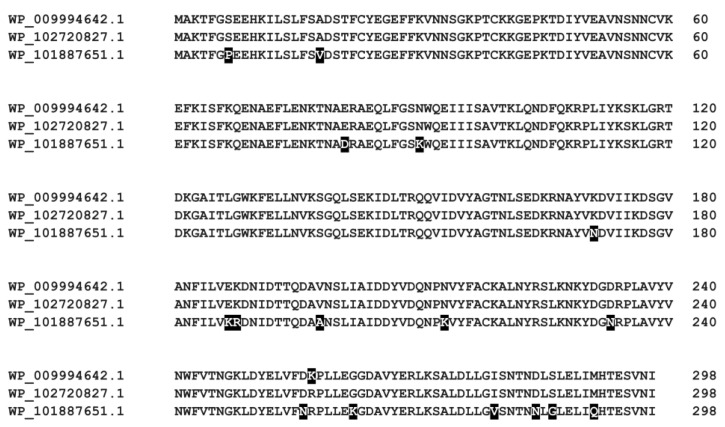
Amino acid sequence alignment for R.Gva14018I and its homologs detected in *Gardnerella* spp. strains. BLASTp (http://blast.ncbi.nlm.nih.gov/) was used for sequence similarity comparisons of R.Gva14018I to NCBI non-redundant protein sequences. The alignment was performed using ClustalW (https://npsa-prabi.ibcp.fr/cgi-bin/npsa_automat.pl?page=npsa_clustalw.html). Nonidentical residues are depicted on a black background. WP_009994642.1 (100% identity) also comprises hypothetical proteins of *Gardnerella* strains ATCC 14019, DSM 4944, GH021, and NCTC10287. WP_102720827.1 (99% identity) indicates hypothetical protein of strain UMB0768. WP_101887651.1 (94% identity) comprises hypothetical proteins of strains DNF01149, UMB0032A, and UMB0032B.

The BLASTp search revealed that several *Gardnerella* spp. strains encode proteins highly similar to R.Gva14018I (100%, 99%, or 94% sequence identity) (Figure 9). The *Gardnerella* spp. strains encoding R.Gva14018I homologs also contain cognate methylases that are highly similar to M.Gva14018I (100% or 99% sequence identity). This indicates that these strains possess a full R-M system. In addition to ATCC 14018, *G. vaginalis* ATCC 14019 and *G. vaginalis* DSM 4944 possess putative REase that recognize GGCC (rebase.neb.com/rebase/rebase.html). Genome analysis revealed a wide diversity of R-M genes at varying frequencies across 106 *Gardnerella* spp. [47]. However, *G. vaginalis* ATCC 49145 and *G. swidsinskii* GV37 with putative R-M genes (rebase.neb.com/rebase/rebase.html; 47) did not produce restriction endonuclease activity in our hands when grown in vitro.

*Gardnerella* species were found not to be defined by the presence or absence of particular R-M systems [47], however, these systems are among the barriers to interspecies and intraspecies HGT [25,48]. Horizontal gene transfer is recognized to be a major force shaping the diversity of *Gardnerella* spp. [13,16,47,49]. More HGT was observed within the same *Gardnerella* species or closely related species (e.g., between *G. piotii* and *G. vaginalis*) than distantly related species/genomospecies, therefore allowing it to maintain species separation in the same vaginal niche [13,47]. *G. vaginalis* and *G. piotii* are the most often isolated species from BV-positive women and those species seem to possess a greater virulence potential relative to *G. leopoldii/G. swidsinskii* [12,15]. *G. vaginalis* has a larger pangenome and the core genome is more recombinant than *G. piotii* that suggest more frequent *G. vaginalis* engagement in HGT [47]. Differences in abundance and target specificity of R-M systems could determine differences in HGT between *G. vaginalis* and *G. piotii.* In this study, cell lysates of 31 *Gardnerella* spp. were tested for the REase activity. Among ten isolates of clade 2 (emended description *G. piotii*, manuscript in preparation), three isolates produced Dam or Dcm methylation-sensitive REases (Figure S2). Highly similar digestion patterns suggest the same specificity of these REases. Among 16 isolates of clade 1 (emended description *G. vaginalis*), including ATCC strains, three strains produced enzymes that cut DNA regardless of methylation. Two strains produced REases with similar DNA digestion patterns; however, none of them matched the DNA digestion pattern of R.Gva14018I, and PCR analysis did not reveal the presence of *gva14018IM* and *gva14018IR* coding genes in these strains. Our data show that the REase activity was more frequently detected among *G. piotii* strains than *G. vaginalis* that may support the potential role of R-M systems as a barrier to HGT in *G. piotii*. Moreover, Type II REases of distinct specificity were limited to the particular *Gardnerella* species. Five isolates of clade 4 (emended description *G. leopoldii/G.swidsinskii*) did not display the REase activity that suggests other barriers and mechanisms of protection against foreign DNA. However, the major limitation to the evaluation of the distribution of Type II REases among species/clades is the small number of strains analyzed in this study. Among limitations is the test for REase activity, which allows the detection of substrate digestion patterns characteristic of Type II REases. *Gardnerella* spp. isolates may produce rare-cutting restriction endonucleases (e.g., Type I or Type III) that require specific DNA substrates to detect their activity. The gene expression behavior of *Gardnerella* spp. depends on the mode of growth, the presence of other BV-associated bacteria, and the host [20]. The expression of genes involved in R-M systems might be affected by the same factors that are not accounted for when cultivating in vitro.

In conclusion, we identified and characterized the R-M system of Type II from the *G. vaginalis* ATCC 14018 strain. R.Gva14018I is a four-base cutter and displays a HaeIII-like restriction endonuclease activity that is widespread in bacteria and archaea. The detected REase activity in *G. vaginalis* and *G. piotii* isolates suggest that Type II R-M systems might constitute the inter-species barriers to HGT. However, the particular role of R-M systems in shaping and maintaining HGT barriers remains to be determined.

## 4. Materials and Methods

### 4.1. Bacterial Strains and Cultivation Conditions

*Gardnerella vaginalis* strains ATCC 14018 and 49145 were purchased from the American Type Culture Collection (ATCC). *Gardnerella* spp. isolates were obtained from previously characterized cultured vaginal samples of women from Lithuania [12]. Frozen bacterial stocks were stored at −80 °C in tryptic soy broth (TSB) (Liofilchem, Roseto degli Abruzzi, Italy) supplemented with 20% (*v*/*v*) horse serum (Oxoid, Thermo Fisher Scientific, Waltham, MA, USA) and 15% (*v*/*v*) glycerol. All *Gardnerella* strains were revived on chocolate agar with Vitox (Oxoid) and incubated at 36 °C under 5% CO_2_ for 48 h. *G. vaginalis* ATCC 14018, ATCC 49145, *Gardnerella* spp. strains [12], and GV37 were grown in 7 mL of liquid sTSB medium (TSB supplemented with 0.15% (*w*/*v*) soluble starch and 2% (*v*/*v*) horse serum) for 24 h, as described previously [15]. Horse serum included into sTSB medium contains nucleases [50]; therefore, unless otherwise indicated, *G. vaginalis* ATCC 14018, ATCC 49145 strains were cultivated in 7 mL of sBHI medium (brain-heart infusion broth (Liofilchem) supplemented with 2% (*w*/*v*) gelatin, 0.5% yeast extract (*w*/*v*), and 0.1% soluble starch (*w*/*v*)) in tightly closed tubes at 36 °C for 22 h. Typically, after cultivation, the ATCC 14018 strain reached OD_600_ = 0.6–0.8 and ATCC 49145 reached OD_600_ = 0.9–1.0. After measurement of the optical density, cells were separated via centrifugation at 13,000× *g* for 2 min, and the supernatants were filtered through 0.1-µm membrane filters (Ultrafree-MC PVDF, Merck Millipore). To assess the endonuclease activity during growth, *Gardnerella* spp. strains were cultivated in 30 mL of sBHI. The samples were collected after incubating for 0, 16, 24, and 44 h at 36 °C. The supernatants were filtered and stored frozen.

*E. coli* strains used in this study are listed in Table S1. LB medium (Thermo Fisher Scientific) was supplemented with the following antibiotics: 100 µg/mL ampicillin for recombinant pJET1.2, pUC57, and pBAD/HisA-based plasmids; 25 µg/mL kanamycin for recombinant pET28a-based plasmids; and 12 or 36 µg/mL chloramphenicol for recombinant pACYC184-based plasmids (Table S2). ArcticExpress (DE3) cultures were incubated overnight in 20 µg/mL gentamicin. Recombinant protein expression in *E. coli* cells harboring prophage DE3 was induced with 0.1 mM isopropyl β-D-1-thiogalactopyranoside (IPTG). Where indicated, the basal expression of recombinant proteins from pET28a(+) [51] and pBAD/HisA [52] plasmids was repressed by adding 0.5% or 1% (*w*/*v*) D(+)-glucose (AppliChem, Merck) to the culture medium.

### 4.2. Amplification of gva14018IR and gva14018IM Genes

A putative R-M system of *G. vaginalis* strain ATCC 14018 is indicated in the REBASE database [35]. Enzymes and kits obtained from Thermo Fisher Scientific were used to manipulate DNA according to standard protocols. The gDNA was extracted using the GeneJET Genomic DNA Purification Kit (#K0721) according to the manufacturer’s recommendations. Genes encoding REase (*gva14018IR*) and MTase (*gva14018IM*) in *Gardnerella* spp. strains were detected with primer pairs GVRe5/GVRe3 and GVMe5/GVMe3 (Table 1). PCR was performed with Platinum II Hot Start Taq polymerase in a 15-µL reaction volume. Amplification reactions included initial denaturation at 94 °C for 2 min, 35 amplification cycles consisting of denaturation for 20 s at 94°C, annealing for 20 s at 43 °C (for *gva14018IR*) or 40 °C (for *gva14018IM*), and extension for 1 min at 72 °C. For cloning, the genes encoding M.Gva14018I and R.Gva14018I were amplified using Phusion Flash II DNA Polymerase and the primer pairs GVMe5/GVMe3, GVRe5/GVRe3, and GvRe5-NheI/GvRe3-XhoI (Table 1). Purified gDNA from the strain ATCC 14018 was used as a template for PCR.

### 4.3. Cloning of the gva14018IM Gene

The DNA fragment coding for M.Gva14018I was cloned in the plasmid pJET1.2/blunt and verified using sequencing. The DNA fragment was excised with Eco105I, and then cloned into the plasmid pACYC184 digested with Eco32I. As a result, the *gva14018IM* gene was inserted downstream of the tetracycline resistance gene promoter. The plasmid was transformed into *E. coli* DH10B, and methyltransferase-positive clones were grown for 18 h. Protection against R.Gva14018RI and BsuRI cleavage was assessed as described in a subsequent section (*MTase activity assay*). The plasmid providing the best protection was named pACYC-Me, and the derived strain harboring this plasmid was named DH10B-Me. Accordingly, *E. coli* strains ER2566 and LMG194 transformed with pACYC-Me were named ER2566-Me and LMG194-Me (Table S1).

The replication origin p15A of the plasmid pACYC-Me was replaced with that of pRSF-Duet1 (Novagen, Merck) to increase the copy number [53] of the *gva14018IM*-containing plasmid. The replication origin of pRSF-Duet-1 was excised with Bsp68I/XbaI, and subsequently cloned into Bst1107I/XbaI–digested pACYC-Me. The selected recombinant plasmid was named pRSFori-Me. Strains DH10B and LMG194 were transformed with pRSFori-Me and assessed for digestion with the *G. vaginalis* 14018 culture supernatant. The derived strains were named DH10B-RSFMe and LMG194-RSFMe.

### 4.4. Cloning the gva14018IR Gene

The DNA fragment amplified with the GvRe5 and GvRe3 primers was cloned into pJET1.2. The resulting plasmid was transformed into strains DH10B and DH10B-Me. Alternatively, the DNA fragment amplified with the GvRe5-NheI and GvRe3-XhoI primers was digested with the respective enzymes and cloned into pET28a(+) and pBAD/HisA. Each resulting plasmid was transformed into DH10B-Me. The thirteen recombinant plasmids from the transformed strains were subjected to sequencing, which revealed the presence of mutations in the *gva14018IR* gene.

Several strategies were used to select the mutation-free *gva14018IR* gene. The amplicon generated with GvRe5-NheI and GvRe3-XhoI primers was cloned into the Eco32I-digested pUC57 vector and transformed into DH10B-Me. IPTG was added to the LB agar plates to induce the lacZ promoter. Five recombinant plasmids were confirmed by sequencing to be free of mutations in *gva14018IR*. The gene was excised with NheI and XhoI, cloned into NheI/SalI-digested pET28a(+), and transformed into *E. coli* ArcticExpress (DE3). The plating medium contained 0.5% (*w*/*v*) glucose. No positive clones were obtained.

The DNA fragment coding for R.Gva14018I was cloned into the NheI/XhoI-digested pBAD plasmid isolated from the DH10B-Me strain. The resulting plasmid was transformed into LMG194-Me, DH10B-Me, and DH10B-RSFMe. The plating medium contained 0–1% (*w*/*v*) glucose. Sequencing results confirmed the presence of mutations in the *gva14018IR* gene.

### 4.5. Expression of R.Gva14018I Mutants in E. coli

Recombinant plasmids bearing either *gva14018IR-4* encoding the truncated protein or *gva14018IR-8* encoding the protein with one amino acid substitution were selected for further experiments. Both fragments were excised from pJET1.2 with NheI/SalI and cloned into respectively digested pET28a(+) to generate N-terminal hexahistidine-tagged proteins. The resulting plasmids bearing *gva14018IR-4* and *gva14018IR-8* were transformed into DH10B. Positive colonies were selected, and the recombinant plasmids were transformed into *E. coli* Tuner (DE3), ER2566, and ArcticExpress (DE3) strains.

### 4.6. Small-Scale Synthesis of Recombinant Proteins

The synthesis of R.Gva14018I-4 and R.Gva14018I-8 in *E. coli* Tuner (DE3), ER2566, and ArcticExpress (DE3) was induced with 0.1 mM IPTG, and then cells were cultivated for 2.5 h at 37 °C, 4 h at 25 °C, or 22–24 h at 12–16 °C, respectively. The cells were harvested and disrupted via sonication (Bandelin Sonopuls HD 3100, Bandelin Electronic, Germany). The soluble fraction was separated, and proteins were purified using the His-Spin Protein Miniprep kit (Zymo Research, Irvine, CA, USA). The insoluble fraction was resuspended in PBS containing 2% SDS and incubated for 10 min at 100 °C. The samples were analyzed by 12% SDS-PAGE under reducing conditions.

### 4.7. Large-scale Protein Synthesis of Gva14018RI-4 and Protein Purification

*E. coli* ArcticExpress (DE3) transformed with pET28 bearing *gva14018IR-4* was cultivated with shaking at 37 °C for 2 h until cultures reached OD_600_ ~ 0.4. The flasks were cooled to 4 °C for 1 h. IPTG was added to a final concentration of 0.1 mM, and cultures were incubated at 12 °C for 24 h with shaking (OD_600_ = 1). The cells were collected by centrifugation at 5000× *g* for 15 min at 4 °C, washed in PBS followed by centrifugation at 3000× *g* for 20 min at 4 °C, and kept frozen at −20 °C. A total of 6 g of wet weight biomass was obtained from 3.6 L culture medium. The cell pellet was suspended in lysis buffer (25 mM Tris-HCI pH 7.4, 0.5% Triton X-100, 0.2 M NaCl, 0.1 M imidazole, 10% glycerol, and 2 mM 2-mercaptoethanol) containing 1 mM phenylmethylsulfonyl fluoride and Halt™ Protease Inhibitor Cocktail (Thermo Fisher Scientific). The cells were incubated at 4 °C for 60 min and then disrupted by sonication. The supernatant was clarified by centrifugation at 30,000× *g* for 25 min. The soluble His-tagged R.Gva14018I-4 protein was purified using Chelating Sepharose Fast Flow with immobilized Ni^2+^ ions (GE Healthcare Bio-Sciences AB, Sweden). The purified protein was dialyzed overnight at 4 °C against buffer (20 mM HEPES pH 7.5, 150 mM NaCl, 5% glycerol, and 1 mM dithiothreitol), and the purified protein was stored at −80 °C. Protein concentration was determined using UV-visual spectrophotometry (calculated extinction coefficient 40,005 M^−1^cm^−1^ at 280 nm) and the standard Bradford method.

### 4.8. Determination of REase Cleavage Site

Plasmid pBR322 was subjected to digestion with cell-free culture supernatant (OD_600_ = 0.6) of *G. vaginalis* strain ATCC 14018. The DNA cleavage products, either untreated or treated with blunting enzyme (CloneJET PCR Cloning kit, Thermo Fisher Scientific), were purified and cloned into pJET1.2 using the CloneJET PCR Cloning kit. The recombinant plasmids were subjected to sequencing. The insert sequences were determined, and the fragment contents were elucidated via alignment with pBR322. The pUC57 plasmid was a template for a single-site PCR substrate generated using primers GV-pUC57-For and GV-pUC57-Rev (Table 1). The obtained DNA fragment of 868 bp was digested with the purified R.Gva14018I-4 protein. The DNA cleavage products were cloned into pJET1.2 and sequenced.

### 4.9. REase Activity Assays

DNA cleavage assays were performed in 18-µL reactions at 37 °C. The following DNA samples were used as substrates: pBR322, pUC57 (both plasmids isolated from *E. coli dam+ dcm+*; Thermo Fisher Scientific), gDNAs (from calf thymus, HCT116 human cell line, and *E. coli*), and a single-site PCR fragment. Assays to detect REase activity in culture supernatants, cell lysates, and crude protein preparations were typically performed in Fast Digest reaction buffer (Thermo Fisher Scientific) containing 0.25 µg pBR322 and 0.1 µg/µL of RNase A. The test sample (2.5 µL) was added to the reaction mixture and incubated for 1–2 h at 37 °C. The reaction was stopped by adding DNA loading dye with SDS (Thermo Fisher Scientific), and the mixture was heated at 65 °C for 10 min before loading on a 1% agarose gel in 1×TAE. The native REase activity in cell-free sTSB medium from *Gardnerella* spp. strains was determined using buffer containing 50 mM Tris-HCl (pH 7.2 at 37 °C) and buffer containing 50 mM sodium acetate (pH 5.0), both supplemented with 1 mM MgCl_2_ and 1 mM CaCl_2_. To compare the specificities of wild-type and recombinant REases, 1 µL of supernatant or 0.7 µg of purified R.Gva14018I-4 was mixed with the substrate (0.25 µg of pUC57 or 0.5 µg of gDNA) in 1× buffer B (10 mM Tris-HCl pH 7.5, 10 mM MgCl_2_, and 0.1 mg/mL BSA) (Thermo Fisher Scientific) containing 0.1 µg/µL of RNase A. The reaction mixture was incubated at 37 °C for 16 h.

The effects of salt concentration, pH, and divalent metal ions were determined using 25 mM Tris-HCl buffer (pH 7.2 at 37 °C) and 25 mM sodium acetate buffer (pH 5.0) containing 0–200 mM NaCl or KCl, 0–15 mM MgCl_2_, 0–10 mM MnCl_2_, CaCl_2_, or NiCl_2_. The cleavage reactions were performed by incubating 1 µg of purified R.Gva14018I-4 with 0.25 µg of pUC57 or 0.4 µg of purified R.Gva14018I-4 with 0.2 µg of single-site DNA fragment for 30 min at 37 °C.

### 4.10. MTase Activity Assay

In vivo DNA methylation by wild-type and recombinant M.Gva14018I was tested by performing the DNA protection assay as follows: 0.5 µg of gDNA was incubated with 1 µL of *G. vaginalis* 14018 culture supernatant (OD_600_ = 0.6) or 0.25 µL BsuRI (Fast Digest, Thermo Fisher Scientific) in the presence of 0.1 µg/µL RNase A in 1× FastDigest (Thermo Fisher Scientific) buffer at 37 °C for 16 h.

### 4.11. Generation of Murine Polyclonal Antibodies Against Recombinant R.Gva14018I-4

Polyclonal antibodies (pAbs) against R.Gva14018I-4 were produced in mice. Animal maintenance and experimental protocols were performed in accordance with FELASA guidelines and Lithuanian and European legislation in the Department of Biological Models (Institute of Biochemistry, Life Sciences Center, Vilnius University). The approval to use mice for immunizations was obtained from the Lithuanian State Food and Veterinary Agency (permission No. G2-117, issued 11 June 2019). Three female 8-week-old BALB/c mice were immunized with 50 µg of recombinant protein R.Gva14018I-4 by subcutaneous injection four times every 28 days. For the first and second immunizations, 50 µg of R.Gva14018I-4 was emulsified with complete (Sigma Aldrich, St. Louis, MO, USA) and incomplete Freund’s adjuvants, respectively. After 94 days, mice were sacrificed, and blood samples were collected. The blood serum was mixed with saturated ammonium sulfate solution to precipitate pAbs.

### 4.12. Immunoblot Detection of R.Gva14018I

*G. vaginalis* strains ATCC 14018 and 49145 were grown for 28 h on chocolate agar with Vitox (Oxoid). The cells were harvested, washed with 0.85% NaCl solution, and kept at −20 °C. The biomass was suspended in PBS containing Halt™ Protease Inhibitor Cocktail and then disrupted by sonication. Cell lysates were clarified by centrifugation at 16,100× *g* for 2 min, and then loaded on a 10% SDS-PAGE gel. After electrophoresis, proteins were transferred to a polyvinylidene difluoride (PVDF) blotting membrane (GE Healthcare Life Science, Freiburg, Germany) under semi-dry conditions. The membrane was blocked with 2% powdered milk (Roth, Germany) in PBS, and then incubated with pAbs against recombinant R.Gva14018I-4. After washing in PBST (PBS supplemented with 0.05% Tween 20), the membrane was incubated with anti-mouse IgG-HRP (Bio-Rad, Hercules, CA, USA) for 1 h. After rinsing, protein bands were developed using 4-chloro-1-naphthol (Sigma Aldrich, St. Louis, MO, USA).

### 4.13. Immunoprecipitation and Mass Spectrometry Analysis of Recombinant R.Gva14018I-4

R.Gva14018I-4 protein was immunoprecipitated with anti-R.Gva14018I-4 pAbs that were coupled to MagnaBind™ Protein A Beads (Pierce Biotechnology, Thermo Fisher Scientific). The pAbs were incubated with magnetic beads in binding buffer (Tris-HCl pH 8.0) with mixing for 1 h. After several washes with binding buffer, the magnetic beads were incubated with R.Gva14018I-4 protein in PBS with mixing for 2 h. After several washes with PBS, the immunocomplexes of pAbs and R.Gva14018I-4 were eluted from the magnetic beads with 0.1 M glycine (pH 3.0). The mixture was supplemented with DTT to a final concentration of 50 mM. Samples were stored at −20 °C. The immunocomplexes were analyzed on an integrated HPLC (Agilent 1290 Infinity)/ESI-MS (Agilent Q-TOF 6520) system equipped with a Poroshell 300SB-C8 column (2.1 × 75 mm, 5 µm) via elution with a linear gradient of solvent A (1% formic acid in water) and B (1% formic acid in acetonitrile).

### 4.14. Bioinformatics Analysis

DNA and protein similarity searches were performed using BLAST (https://blast.ncbi.nlm.nih.gov/Blast.cgi). The HHpred server [41] (https://toolkit.tuebingen.mpg.de/tools/hhpred) was used for structural similarity searches. Secondary structure predictions were performed using the PSIPRED server (http://bioinf.cs.ucl.ac.uk/psipred) [42]. The PDEXK recognition server (http://bioinformatics.ibt.lt/pdexk) [39] was implemented to identify nucleases of the PD-(D/E)XK superfamily.

## Figures and Tables

**Table 1:** Oligonucleotides used in this study.

Primer Name	Primer Sequence 5′→3′	T Annealing (°C)	Amplicon Size (bp)
GvMe5	TACGTAATGAAGGTATTAAGCTTATTTAG	56	1024
GvMe3	TACGTACTTATCTGAATTATGATAGAACT		
GVRe5	GCTAGCATGGCAAAAACATTTGGTT	58	917
GVRe3	GTCGACATCTATGCCTATATGTTCACAG		
GvRe5-NheI	GTCTAGCTAGCATGGCAAAAACATTTGGTT	55	917
GvRe3-XhoI	GATCTCGAGTTATATGTTCACAGATTCAGT		
GV-pUC57-For	CCGCGTTGCTGGCGTTTTTC	65	868
GV-pUC57-Rev	GTAGTTATCTACACGACGGGGAGTC		

## Supplementary Materials

The following are available online at https://www.mdpi.com/2076-0817/9/9/703/s1, Figure S1: Enzymatic activity in the cell-free culture supernatants from *G. vaginalis* ATCC 14018 at various time points during cultivation, Figure S2: REase activity in the clarified whole cell lysates of *Gardnerella* spp. strains 47.3, 63.2, 78.1, 86.3, 105.1, *G. vaginalis* ATCC 49145 and ATCC 14018, Figure S3: Detection of the *gva14018IR* (A) and *gva14018IM* (B) genes in *Gardnerella* spp. isolates by gene-specific PCR with the primer pairs GVRe5/GVRe3 and GVMe5/GVMe3, Figure S4: Molecular mass determination of R.Gva14018I-4 by mass spectrometry, Figure S5: Effects of cofactors, pH and salt concentration on the R.Gva14018I-4 activity, Table S1: Bacterial strains used in this study, Table S2: Plasmids used in this study.
