# Supplementary material for: Type II Restriction-Modification System from Gardnerella vaginalis ATCC 14018
Source: Pathogens. 2020 Aug 27;9(9):703. doi: 10.3390/pathogens9090703 (PMC7559349; doi:10.3390/pathogens9090703)
Supplement: Supplementary file 1 [file pathogens-09-00703-s001.zip › pathogens-870488-Supplementary - Final Version/Figure S1.pdf]

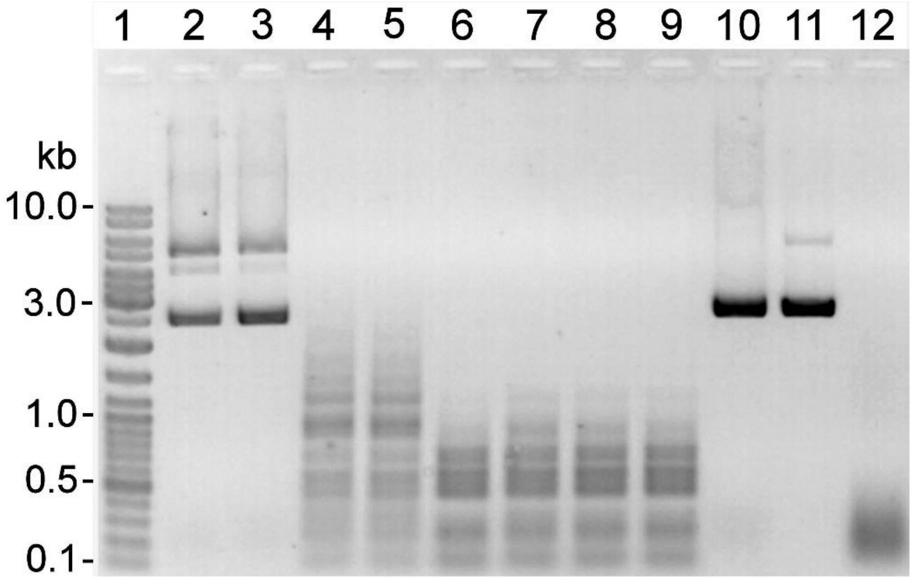

Figure S1. Enzymatic activity in the cell-free culture supernatants from *G. vaginalis* ATCC 14018 at various time points during cultivation. pBR322 was incubated with samples taken from duplicate cultures after 0 h of cultivation, OD_{600} = 0 (lanes 2, 3), 16 h at OD_{600} = 0.019 of the first culture and OD_{600} = 0.013 of the second culture (lanes 4, 5), 24 h, OD_{600} = 0.380, 0.377 (lanes 6, 7), and 44 h, OD_{600} = 0.300, 0.283 (lanes 8, 9). Lane 1: Gene Ruler™ Ladder Mix (Thermo Fisher Scientific); lane 10: pBR322 incubated with sBHI medium, lane 11: untreated pBR322; lane 12: pBR322 treated with *S. pneumoniae* culture supernatant.
