# Supplementary material for: Type II Restriction-Modification System from Gardnerella vaginalis ATCC 14018
Source: Pathogens. 2020 Aug 27;9(9):703. doi: 10.3390/pathogens9090703 (PMC7559349; doi:10.3390/pathogens9090703)
Supplement: Supplementary file 1 [file pathogens-09-00703-s001.zip › pathogens-870488-Supplementary - Final Version/Figure S2.pdf]

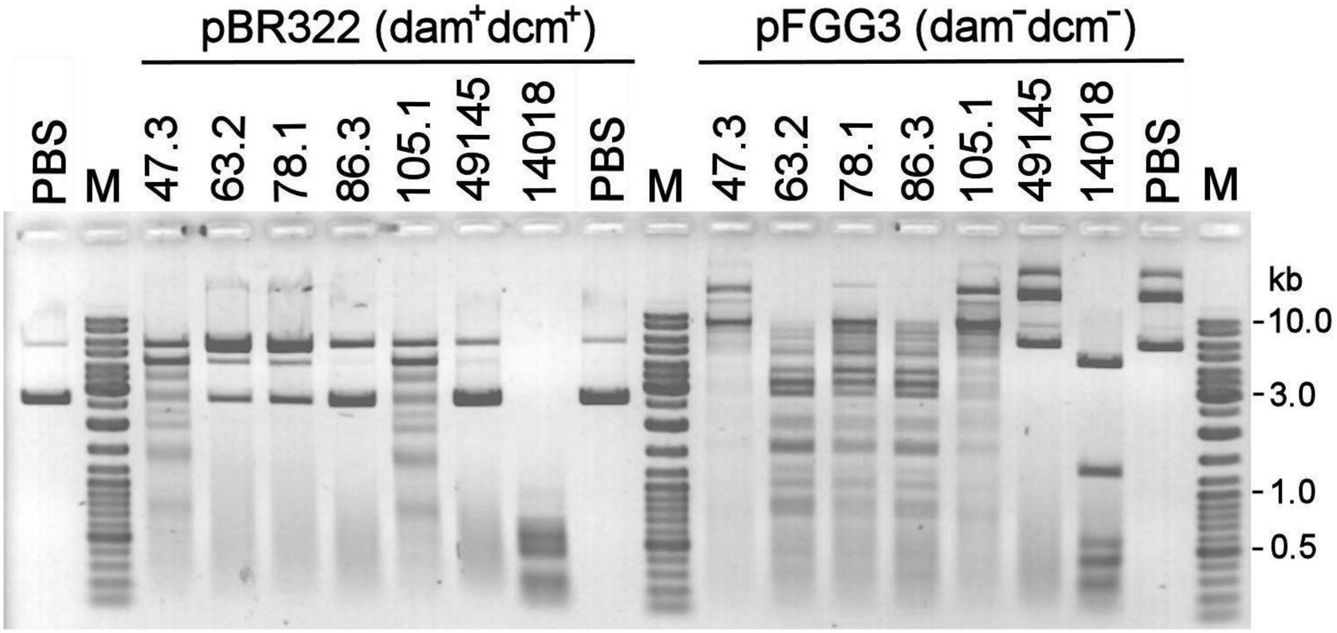

Figure S2. REase activity in the clarified whole cell lysates of *Gardnerella* spp. strains 47.3, 63.2, 78.1, 86.3, 105.1, *G. vaginalis* ATCC 49145, and ATCC 14018. *Gardnerella* spp. were cultivated in sTSB medium as described in *Methods*. Isolates 47.3 (after cultivation OD_{600}=0.58) and 105.1 (OD_{600}=0.51) belong to clade 1, isolates 63.2 (OD_{600}=0.76), 78.1 (OD_{600}=0.56), and 86.3 (OD_{600}=0.75) belong to clade 2. After cultivation *G. vaginalis* ATCC 14018 strain reached OD_{600}=0.65, *G. vaginalis* ATCC 49145 reached OD_{600}=0.98. Cells were resuspended in PBS containing Halt™ protease inhibitor cocktail (Thermo Fisher Scientific) and disrupted by sonication. Plasmids pBR322 and pFGG3 (Table S2) were incubated with 2.5 µl of cell lysate in Fast Digest buffer (Thermo Fisher Scientific) containing RNase for 2 h 20 min at 37°C. pBR322 was isolated from *E. coli dam*$^{+}$ *dcm*$^{+}$ (Thermo Fisher Scientific) and pFGG3 was isolated from *E. coli* GM119 *dam*$^{-}$ *dcm*$^{-}$ (Table S1). Lane PBS: plasmids pBR322 and pFGG3 treated with PBS.
