# Supplementary material for: Type II Restriction-Modification System from Gardnerella vaginalis ATCC 14018
Source: Pathogens. 2020 Aug 27;9(9):703. doi: 10.3390/pathogens9090703 (PMC7559349; doi:10.3390/pathogens9090703)
Supplement: Supplementary file 1 [file pathogens-09-00703-s001.zip › pathogens-870488-Supplementary - Final Version/Figure S3.pdf]

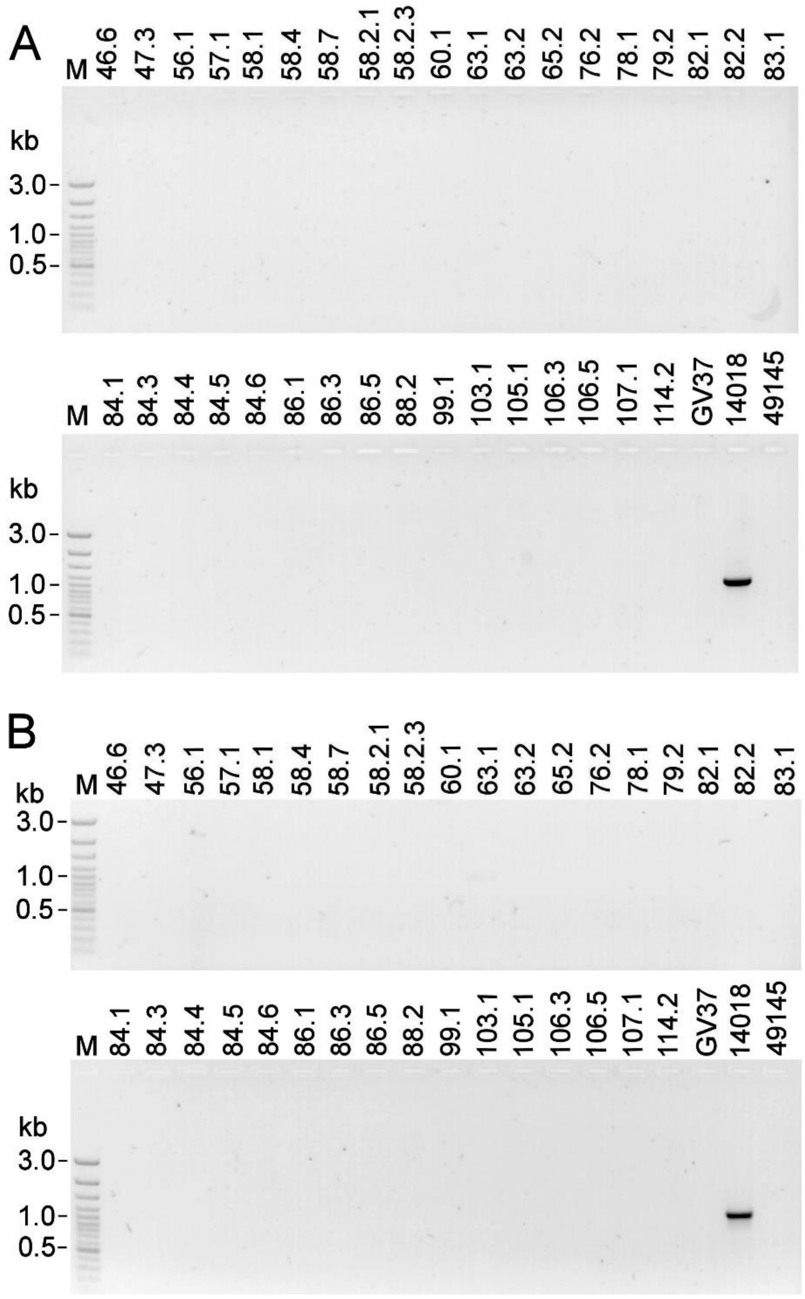

Figure S3. Detection of the *gva14018IR* (A) and *gva14018IM* (B) genes in *Gardnerella* spp. isolates by gene-specific PCR with the primer pairs GVRe5/GVRe3 and GVMe5/GVMe3, respectively. Lane M: Gene Ruler 100bp Plus DNA Ladder (Thermo Fisher Scientific).
