# Supplementary material for: Type II Restriction-Modification System from Gardnerella vaginalis ATCC 14018
Source: Pathogens. 2020 Aug 27;9(9):703. doi: 10.3390/pathogens9090703 (PMC7559349; doi:10.3390/pathogens9090703)
Supplement: Supplementary file 1 [file pathogens-09-00703-s001.zip › pathogens-870488-Supplementary - Final Version/Figure S4.pdf]

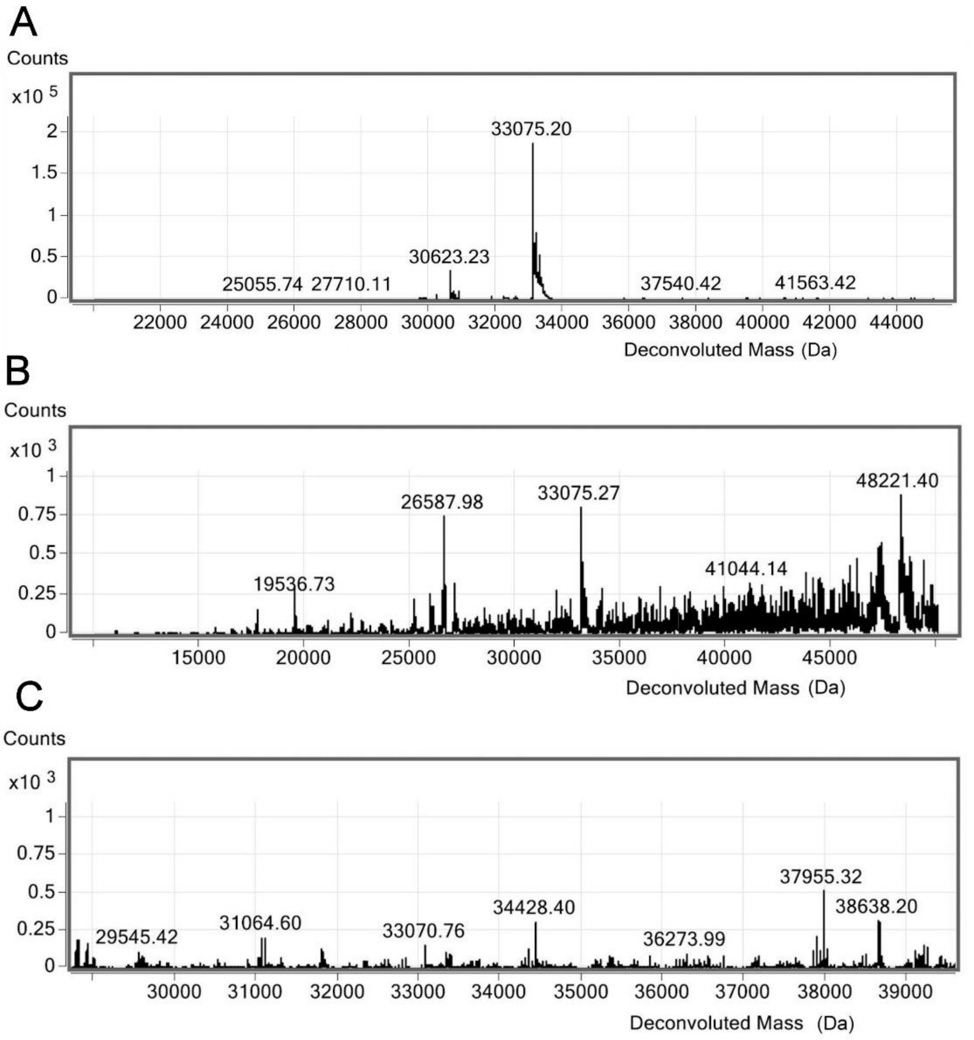

Figure S4. Molecular mass determination of R.Gva14018I-4 by mass spectrometry. (**A**) Purified R.Gva14018I-4. (**B**) R.Gva14018I-4 (0.1µg) immunoprecipitated with specific pAbs (2.23 µg). (**C**) Anti-R.Gva14018I-4 pAbs.
