# Supplementary material for: Type II Restriction-Modification System from Gardnerella vaginalis ATCC 14018
Source: Pathogens. 2020 Aug 27;9(9):703. doi: 10.3390/pathogens9090703 (PMC7559349; doi:10.3390/pathogens9090703)
Supplement: Supplementary file 1 [file pathogens-09-00703-s001.zip › pathogens-870488-Supplementary - Final Version/Figure S5.pdf]

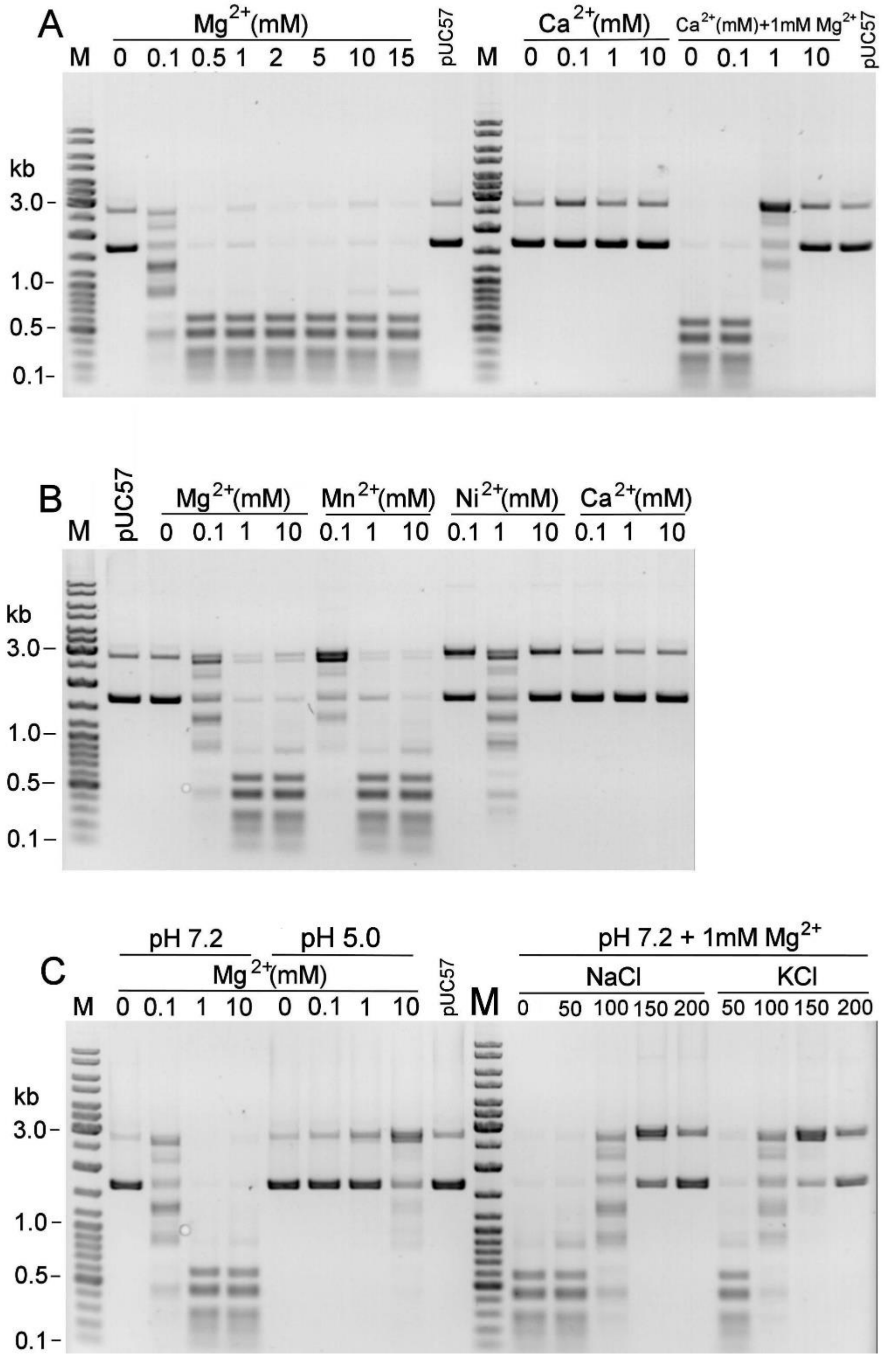

Figure S5. Effects of cofactors, pH and salt concentration on R.Gva14018I-4 activity. (**A**) Plasmid pUC57 was digested with purified R.Gva14018I-4 in the presence of increasing amounts of Mg^{2+} and Ca^{2+}as described in Methods. R.Gva14018I-4 was incubated with pUC57 in reactions supplemented with 1 mM Mg^{2+} at increasing amounts of Ca^{2+}. (**B**) pUC57 cleavage in the presence of increasing amounts of Mg^{2+}, Mn^{2+}, Ni^{2+} and Ca^{2+}. (**C**) pUC57 was digested with purified R.Gva14018I-4 in the presence of increasing amounts of Mg^{2+} at pH 7.2 and pH 5.0 as described in Methods. The reactions were supplemented with 1 mM Mg^{2+} to test the effects of salt concentration at pH 7.2. Lane pUC57 in A, B indicates the plasmid with no enzyme added. Lane M: Gene Ruler™ Ladder Mix (Thermo Fisher Scientific).
