# Supplementary material for: Type II Restriction-Modification System from Gardnerella vaginalis ATCC 14018
Source: Pathogens. 2020 Aug 27;9(9):703. doi: 10.3390/pathogens9090703 (PMC7559349; doi:10.3390/pathogens9090703)
Supplement: Supplementary file 1 [file pathogens-09-00703-s001.zip › pathogens-870488-Supplementary - Final Version/Table S1.pdf]

Table S1. Bacterial strains used in this study

Strain	Genotype or purpose	Reference or source
DH10B	F^{-} *mcr*A Δ(*mrr*-*hsd*RMS-*mcr*BC) φ80*lac*ZΔM15 Δ*lac*X74 *rec*A1 *end*A1 *ara*D139 Δ(*ara*-*leu*)7697 *gal*U *gal*K λ^{-} *rps*L(Str^{R}) *nup*G	Thermo Fisher Scientific
DH10B-Me	Methylation-proficient DH10B carrying plasmid pACYC-Me	This study
DH10B-RSFMe	Methylation-proficient DH10B carrying plasmid pRSFori-Me	This study
Tuner(DE3)	F^{-} *ompT hsdS*$_{B}$ (r_{B}^{-} m_{B}^{-}) *gal dcm lacY1*(DE3)	Novagen, Merck, USA
ER2566	*fhu*A2 *lac*Z::T7 *gene1* [*Ion*] *omp*T *gal sul*A11 R(*mcr*-73::miniTn10-Tet^{S})2 [*dcm*] R(*zgb*-210::Tn10-Tet^{S}) *end*A1 Δ(*mcr*C-*mrr*)114::IS10	New England Biolabs, USA
ER2566-Me	Methylation-proficient ER2566 carrying plasmid pACYC-Me	This study
ArcticExpress(DE3)	*E. coli* B F^{-} *ompT hsdS*(r_{B}^{-} m_{B}^{-}) *dcm*$^{+}$Tet^{r}*gal* λ(DE3) *endA* Hte [*cpn10 cpn60* Gent^{r}]	Agilent Technologies, USA
LMG194	F^{-} Δ*lac*X74 *gal*E *thi rps*L(Str^{R}) Δ*pho*A (*Pvu*II) Δ*ara*714 *leu*::Tn*10*	Thermo Fisher Scientific
LMG194-Me	Methylation-proficient LMG194 carrying plasmid pACYC-Me	This study
LMG194-RSFMe	Methylation-proficient LMG194 carrying plasmid pRSF ori-Me	This study
GM119	F- *sup*E44 *lac*Y1 *gal*K2 *gal*T22 *met*B1 *dam*-3 *dcm*-6 *tsx*-78 *lambda*-	[1]

1. Arraj, J. A.; Marinus, M. G. Phenotypic reversal in *dam* mutants of *Escherichia coli* K-12 by a recombinant plasmid containing the *dam*$^{+}$ gene. *J. Bacteriol.* **1983**, *153*, 562–565.
