# Supplementary material for: Type II Restriction-Modification System from Gardnerella vaginalis ATCC 14018
Source: Pathogens. 2020 Aug 27;9(9):703. doi: 10.3390/pathogens9090703 (PMC7559349; doi:10.3390/pathogens9090703)
Supplement: Supplementary file 1 [file pathogens-09-00703-s001.zip › pathogens-870488-Supplementary - Final Version/Table S2.pdf]

Table S2. Plasmids used in this study

Plasmid	Genotype or purpose	Reference or source
pBR322	Substrate for REase activity assay	Thermo Fisher Scientific
pFGG3	Substrate for REase activity assay	[1]
pUC57	Ap^R; pMB1 replication origin; cloning vector / REase activity assay substrate	Thermo Fisher Scientific
pJET1.2	Ap^R; pMB1 replication origin; cloning vector	Thermo Fisher Scientific
pET28a(+)	Km^R; pMB1 replication origin; expression vector	Novagen, Merck
pBAD/HisA	Ap^R; pMB1 replication origin; expression vector	Thermo Fisher Scientific
pACYC184	Tc^R, Cm^R; p15A replication origin; expression vector for *gva14018IM*	New England Biolabs, USA
pRSF-Duet-1	Km^R; RSF1030 replication origin donor	Novagen, Merck
pACYC-Me	Cm^R; pACYC184 carrying *gva14018IM*	This study
pRSFori-Me	Cm^R; RSF1030 replication origin substituted for p15A in pACYC-Me	This study

1. Slibinskas, R.; Samuel, D.; Gedvilaite, A.; Staniulis, J.; Sasnauskas, K. Synthesis of the measles virus nucleoprotein in yeast *Pichia pastoris* and *Saccharomyces cerevisiae*. *J Biotechnol* **2004**, *107*, 115–124, doi:10.1016/j.jbiotec.2003.10.018.
